# Lumbar hernia misdiagnosed as a subcutaneous lipoma: a case report

**DOI:** 10.1186/1752-1947-3-9322

**Published:** 2009-12-10

**Authors:** Giuseppe Pietro Mingolla, Gianfranco Amelio

**Affiliations:** 1 Department of General Surgery, S.M. della Scaletta Hospital, via Montericco n 4, 40026 Imola, Italy

## Abstract

**Introduction:**

Lumbar hernia is a rare abdominal wall defect and clinical suspicion is necessary for diagnosis.

**Case presentation:**

We report the case of a 40-year-old Caucasian woman with a superior lumbar hernia (Grynfeltt hernia) initially misdiagnosed as a recurrent lipoma. The correct diagnosis was made intra-operatively and the hernia was repaired using synthetic mesh. The patient was free of recurrence at 4 months after the operation.

**Conclusion:**

A lumbar or flank mass should always raise suspicion of a lumbar hernia. Ultrasound and computed tomography may confirm the diagnosis. Adequate surgical treatment should be planned on the basis of etiology and hernia size. Both open and laparoscopic techniques can be used with good results.

## Introduction

Lumbar hernias are rare defects involving two weak areas of the posterolateral abdominal wall: the superior lumbar triangle of Grynfeltt, which is the most common site, and the inferior lumbar triangle of Petit. In large congenital or postsurgical hernias the defect wall can affect the entire lumbar region. Lower-back pain is the most common symptom although small hernias may be asymptomatic except for a palpable mass. Misdiagnosis with subcutaneous lipoma is possible. Adequate surgical treatment depends largely on the type and size of the hernia and both open and laparoscopic techniques can be used with good results.

## Case presentation

A 40-year-old Caucasian woman presented with a diagnosis of a recurrent lipoma of the lumbar region. She had a palpable mass and mild occasional pain at the same site where she had been submitted elsewhere to ambulatory excision of a lipoma. An ultrasound examination confirmed the presence of a subcutaneous lipoma. The surgeon who treated the patient omitted a careful physical examination and an excision of the suspected recurrent lipoma was planned under local anesthesia in our day surgery unit.

**Figure 1:**
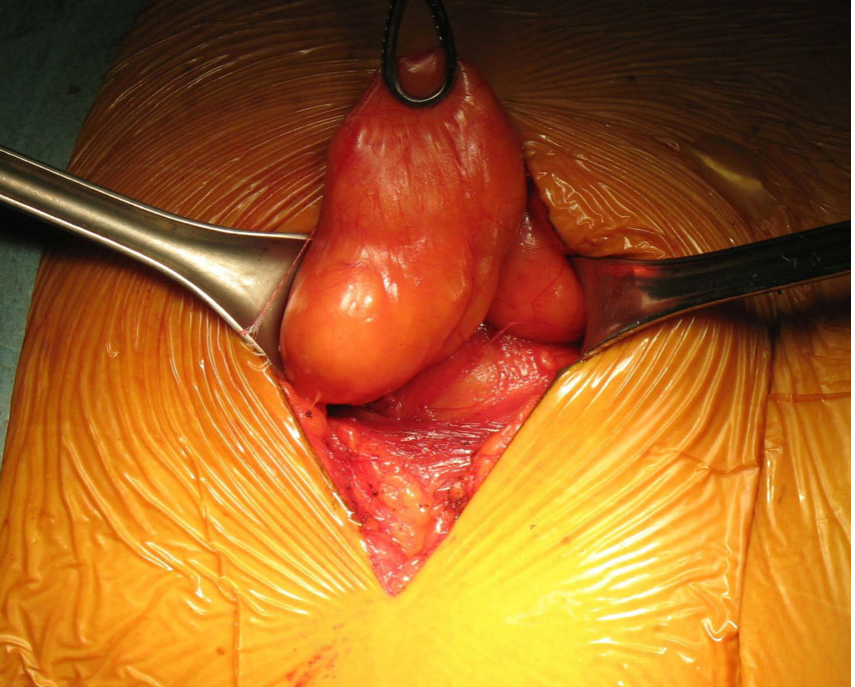
**Herniated fatty mass through a parietal defect**.

**Figure 2:**
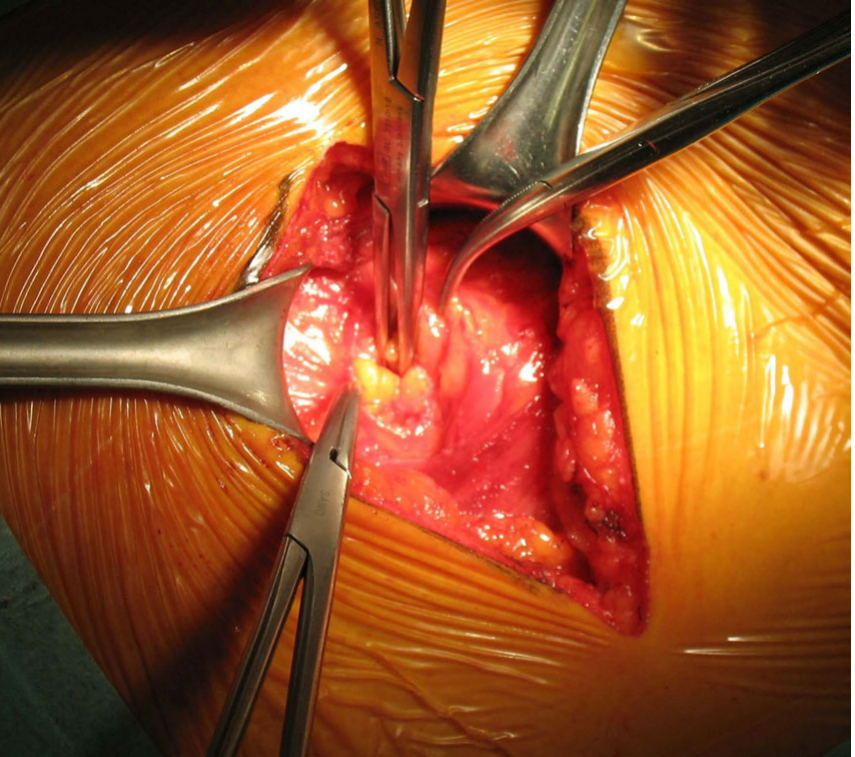
**The parietal defect**.

**Figure 3:**
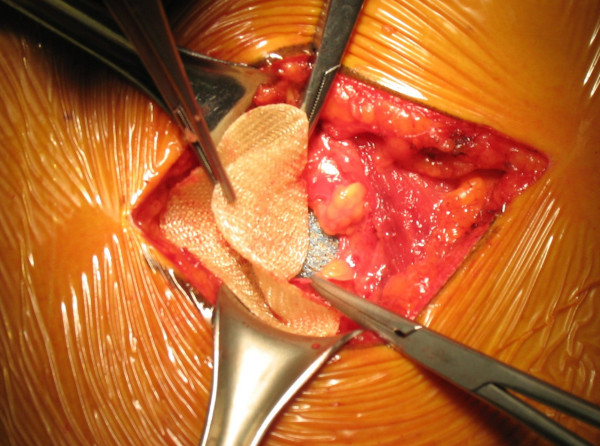
**The plug is anchored in the preperitoneal space**.

**Figure 4:**
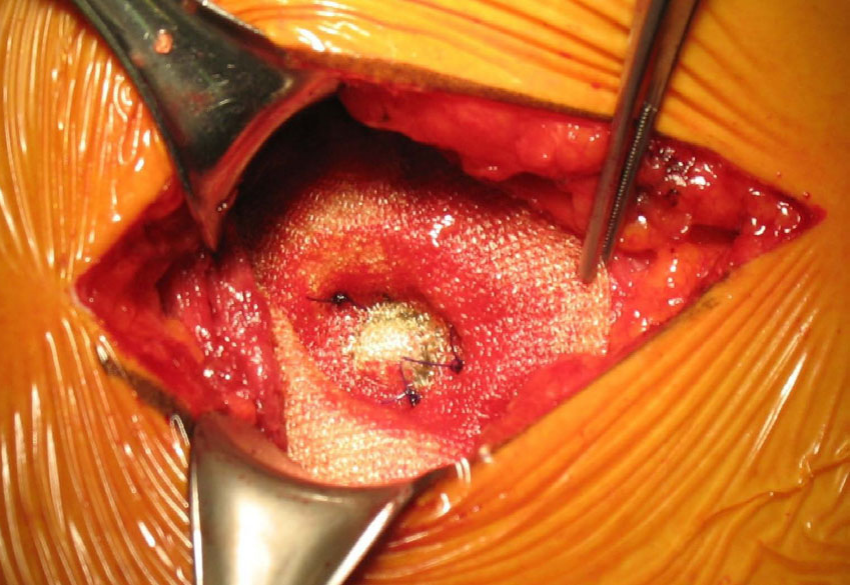
**The plug is fixed with sutures to the muscles**.

During the intervention, the aspect of the mass was clearly atypical of a lipoma and the suspicion of a lumbar hernia emerged (Figure 1). The herniated fatty mass was isolated and reduced in the abdomen. The abdominal wall defect was 3 cm in diameter (Figure 2), and was repaired using a medium-sized Ultrapro plug (Ethicon) which was anchored in the preperitoneal space and fixed with sutures to the muscles using a tension-free technique (Figure 3 and Figure 4). The intervention was completed with the support of target controlled infusion with propofol.

Her postoperative course was uneventful and she was discharged on the same day. Follow-up at 4 months did not show recurrence.

## Discussion

Lumbar hernias are rare and a recent review reported approximately 300 cases [1]. They are classified as congenital, generally associated with other malformations, or acquired, manifesting in adults spontaneously or secondary to trauma or surgical incision [2].

Lumbar hernia may occur in two weak areas of the posterolateral abdominal wall: the superior lumbar triangle of Grynfeltt, which is the more common site, and the inferior lumbar triangle of Petit. In large hernias the defect wall can affect all of the lumbar region [3,4].

Symptomatology frequently consists of only lower back pain. Small hernias may be asymptomatic except for a palpable mass. In less than 10% of cases, the onset is acute with bowel obstruction [5,6].

Anamnesis is helpful for diagnosis in post-traumatic or postsurgical lumbar hernias while in spontaneous adult hernias, misdiagnosis may occur [1,7].

Clinical suspicion is fundamental to guide imaging diagnosis because extraperitoneal fat herniated through a wall defect may mimic a lipoma. Computed tomography (CT) or magnetic resonance imaging (MRI) in patients with a suspected hernia can confirm the diagnosis adding information on parietal defect size, hernia content and muscular trophism [8].

Adequate surgical treatment depends largely on the type and size of the hernia. A single surgeon cannot gain great experience in this pathology but knowledge gained in treatment of other abdominal wall hernias helps in proper planning of surgery. Both open and laparoscopic techniques can be used with good results [9].

In small defects as present in our patient, the anterior approach is easy and effective; we used a plug with an anchor that provides stability positioned through the fascial defect in the preperitoneal space, and the body and rim can be sutured to muscles. Anterior repair is appropriate for repairing recurrent or large defects with a double mesh or a gluteus aponeurosis flap [10]. Laparoscopic repair has been used successfully in different reports with less pain, shortened hospital stay and good cosmetic and functional results [11-13].

## Conclusion

Although a rare pathology, knowledge of lumbar hernia is important to avoid misdiagnosis. In particular, a lumbar or flank mass should always raise suspicion of a lumbar hernia. Ultrasound and CT may confirm the diagnosis. Appropriate surgical treatment should be planned on the basis of etiology and hernia size.

## Abbreviations

CT: computed tomography; MRI: magnetic resonance imaging.

## Consent

Written informed consent was obtained from the patient for publication of this case report and any accompanying images. A copy of the written consent is available for review by the Editor-in-Chief of this journal.

## Competing interests

The authors declare that they have no competing interests.

## Authors' contributions

GPM and GA performed the operation. GPM was a major contributor in writing the article and reviewed the relative literature. All authors read and approved the final manuscript.
